# Gustatory Function and the Uremic Toxin, Phosphate, Are Modulators of the Risk of Vascular Calcification among Patients with Chronic Kidney Disease: A Pilot Study

**DOI:** 10.3390/toxins12060420

**Published:** 2020-06-25

**Authors:** Shih-I Chen, Chin-Ling Chiang, Chia-Ter Chao, Chih-Kang Chiang, Jenq-Wen Huang

**Affiliations:** 1 Nephrology Division, Department of Internal Medicine, National Taiwan University Hospital Beihu Branch, Taipei 108, Taiwan; csi0604@yahoo.com.tw; 2 Geriatric and Community Medicine Research Center, National Taiwan University Hospital BeiHu Branch, Taipei 108, Taiwan; 3 Department of Nursing, National Taiwan University Hospital Beihu Branch, Taipei 108, Taiwan; a221922519@gmail.com; 4 Graduate Institute of Toxicology, National Taiwan University, Taipei 10617, Taiwan; ckchiang@ntu.edu.tw; 5 Department of Integrative Diagnostics and Therapeutics, National Taiwan University Hospital, Taipei 100, Taiwan; 6 Nephrology Division, Department of Internal Medicine, National Taiwan University Hospital Yunlin Branch, Yunlin County 260, Taiwan; 007378@ntuh.gov.tw

**Keywords:** aortic calcification, chronic kidney disease, gustation, nutrition, taste dysfunction, vascular calcification

## Abstract

Patients with chronic kidney disease (CKD) have an increased risk of vascular calcification (VC), including aortic arch calcification (AAC). Few investigated the influence of gustatory function on the probability of having VC. We examined whether gustatory function results modulated the probability of having VC in patients with CKD. We prospectively enrolled adults with CKD (estimated glomerular filtration rate <60 mL/min/1.73 m^2^), with their AAC rated semi-quantitatively and gustatory function assessed by objective and subjective approaches. Multiple logistic regression was used to analyze the relationship between gustatory function results and AAC. Those with AAC had significantly better objective gustatory function in aggregate scores (*p* = 0.039) and categories (*p* = 0.022) and less defective bitter taste (*p* = 0.045) and scores (*p* = 0.037) than those without. Multiple regression analyses showed that higher aggregate scores (odds ratio (OR) 1.288, *p* = 0.032), or better gustatory function, and higher bitter taste scores (OR 2.558, *p* = 0.019) were each associated with a higher probability of having AAC among CKD patients; such an association was modulated by serum phosphate levels. In conclusion, better gustatory function was independently correlated with having AAC among CKD patients. A follow-up of VC severity may be an underrecognized component of care for CKD patients with a preserved gustatory function.

## 1. Introduction

Vascular calcification (VC) is a dreadful complication responsible for the increased risk of cardiovascular events among patients with chronic kidney disease (CKD) or end-stage renal disease (ESRD). In the forms of coronary artery calcification, aortic arch calcification (AAC) or abdominal aortic calcification, VC impairs myocardial perfusion, increases cardiac afterload and blood pressure (BP) from aortic stiffening and compromises visceral blood flow, leading to ischemia involving vital organs [1]. Despite the observed risk associated with VC, currently, there is a paucity of available therapies proven to be effective against this disease [2], which is attributed to the incomplete understanding of the pathogenesis of uremic VC.

The pathophysiology of uremic VC includes, but is not limited to, passive mineral deposition and active osteoid matrix deposition by phenotypically altered vascular smooth muscle cells (VSMCs) with osteoblast-like activity, propagated by calciprotein or procalcific microvesicles release, the suppression of anticalcific effectors and epigenetic dysregulation [3,4]. Among these intertwining processes, phosphate appears to be a vital contributor to uremic VC, as hyperphosphatemia has been shown to accelerate VSMC trans-differentiation and directly participates in calcium-containing osteoid matrices deposition within vascular media [5,6]. The treatment of VSMCs with inorganic phosphate donors is also a well-established in vitro model mimicking VC, lending support to the significance of this phosphate-VC association [6]. Strategies aiming to decrease phosphate exposure and lower serum phosphate levels can be indispensable if we wish to ameliorate the extent of uremic VC for CKD/ESRD patients [7]. Moreover, serum phosphate is mainly influenced by dietary factors and how these patients determine the types, the ingredients and the amount of food ingested has a major impact on their cumulative phosphate load and subsequent serum phosphate levels [8]. Sergi et al. reported that taste loss in older adults potentially led to a preferential consumption of foods with sweet and salty flavors [9]. Increased intakes of these foods, along with a greater taste intensity for phosphate-containing salts in older adults with CKD [10], jointly contributes to an increased phosphate ingestion and, thereby, higher serum phosphate levels. From this perspective, an individual’s taste integrity, or gustatory function, can be a potential modifier of serum phosphate levels, although this issue remains under-addressed.

Gustatory function is an integral component that assists us in detecting flavors, in addition to the olfactory and trigeminal sensations. Deranged gustatory function has been associated with a higher risk of developing metabolic disorders such as obesity [11]. Patients with renal function impairment have a significantly higher probability of acquiring dysgeusia and an altered gustatory threshold compared to those without [12], and this abnormality compromises their quality of life and potentially contributes to nutritional imbalances. However, it is still unclear whether gustatory function plays a role in influencing the probability of having VC and whether such a relationship is affected by serum phosphate levels. We hypothesized that results from gustatory function tests in patients with CKD could modulate the probability of having VC, and the relationship might be altered by serum phosphate levels. We examined this issue using a prospectively recruited group of patients with non-dialysis CKD.

## 2. Results

During the study period, we screened through 80 non-dialysis CKD patients—among whom, 2 and 20 were excluded for an inability to follow study procedures and the lack of AAC results, respectively, leaving 58 participants with CKD for analysis. Their mean age was 72.4 ± 10.9 years, with a male predominance (65.5%), and over half of them had hypertension (81%) and diabetes mellitus (DM) (53.5%) (Table 1).

Those with AAC had significantly higher ages (*p* = 0.03) than those without, while there was a trend toward a higher prevalence of hypertension (*p* = 0.073), DM (*p* = 0.079) and having a higher serum phosphate (*p* = 0.082) level in the former group compared to the latter (Table 1). On the other hand, there was no significant difference between CKD patients with and without AAC regarding other comorbidities, anthropometric parameters and laboratory data, including hemogram, lipid profile, renal function and other electrolyte levels, except a trend of higher serum phosphate in those with AAC (Table 1).

### 2.1. Comparison of Nutrition, Appetite and Gustatory Function Test Results between CKD Patients with and without VC

We further examined the subjective and objective nutritional status, appetite and gustatory function results between participants with and without AAC (Table 2). The Mini-Nutritional Assessment (MNA) and the Council on Nutrition Appetite Questionnaire (CNAQ) scores did not differ between CKD patients with and without AAC (Table 1). CKD patients with AAC had significantly better objective gustatory function, manifesting as a higher proportion of increasing taste strip aggregate scores (*p* = 0.039) and a greater number of intact taste categories (*p* = 0.046) than those without (Table 2). Specifically, those with AAC were significantly more likely to have high bitter taste scores than those without (*p* = 0.045), while those without AAC were more likely to have defective bitter tastes than those with (*p* = 0.045) (Table 2). There were no differences regarding the other three taste categories (sour, salty and sweet) between those with and without AAC. Finally, the presence of AAC among CKD patients did not influence the subjective perception of taste intactness and taste importance (Table 2).

### 2.2. Investigating the Relationship between Gustatory Function Results, other Clinical Features and VC among CKD Patients

We next conducted multiple regression analyses to examine the relationship between gustatory function results and AAC in these participants. Based on univariate analysis findings and due to the modest cohort size, we selected age, gender, DM, hypertension and individual items of gustatory function results (“the basic model”) in the multiple regression analyses. Advanced age and having hypertension were significantly associated with having AAC across all models tested, which was compatible with results from the existing literature [13,14] (Table 3). Interestingly, higher aggregate taste strip scores, or a better gustatory function, were significantly associated with a higher probability of having AAC among CKD patients (odds ratio (OR) 1.288, 95% confidence interval ([CI) 1.022–1.623), while having more intact taste category counts also showed such effects (OR 1.923, 95% CI 1.152–3.211) (models 1 and 3; Table 3). Better bitter taste scores (OR 2.558, 95% CI 1.167–5.606) and a lower proportion of having defective bitter taste (OR 0.175, 95% CI 0.036–0.847) were also correlated with an increased probability of having AAC among CKD patients (models 5 and 7; Table 3). The variance inflation factors (VIFs) for these variables in regression analyses were all less than 2, precluding the existence of collinearity. Furthermore, if we added serum phosphate levels into the regression models, a higher serum phosphate replaced the gustatory function parameters as the independent factor in models incorporating intact taste category counts or bitter taste scores but not others (models 2, 4, 6 and 8; Table 3).

## 3. Discussion

In this cross-sectional study, we discovered that objective gustatory function results, especially those related to the bitter taste, positively correlated with the probability of having uremic VC among a group of CKD patients. This relationship was partially explained for by higher serum phosphate levels, but there seemed to be other unrecognized factors that also accounted for this association. This phenomenon is interesting, and further studies may be needed to affirm our findings.

The assessment of the gustatory function can be divided into subjective and objective approaches, but findings from these two approaches may have different clinical implications. For example, Hunt et al. showed that only 10% to 25% of patients with a subjective complaint of taste abnormality had objective gustatory impairment, while they might have olfactory dysfunction instead [15]. Furthermore, prior reports suggested the possibility that a subjective gustatory function might be partially reflective of changes in subjective appetite, oral cavity intactness and overall self-rated health status [16], whose clinical meaning may differ substantially from those of the objective gustatory function results. In this study, we discovered that there were no significant differences in the subjective gustatory function results between those with and without VC (Table 2).

The etiologies of gustatory dysfunction are multiple and can be divided mechanistically into several categories, including transport disorders and sensory and neuronal deficits [17]. Transport disorders, such as salivary dysfunction and oropharyngeal infections, involves illnesses involving the relay of taste stimuli to the receptors, while sensory and neuronal deficits result from injuries to gustatory organs and peripheral/central nervous systems, respectively. Patients with CKD may be at-risk of developing gustatory dysfunction related to all these mechanisms, since the accumulation of uremic toxins can contribute to uremic neuropathy and encephalopathy [18]. However, in this study, we could not differentiate between the origins of gustatory dysfunction among participants.

Mechanisms behind the association between a better objective gustatory function and having uremic VC can involve multiple aspects. It is intuitive that patients with CKD and a preserved gustatory function may ingest more food compared to those with an impaired one, and the increased food consumption leads to a greater production of uremic solutes, leading to a greater risk of VC. This is supported by the influences on the probability of having AAC posed by serum phosphate in our regression models (Table 3). There are other uremic toxins that potentially aggravate the risk of VC among CKD/ESRD patients, including protein-bound ones (indoxyl sulfate and *p*-cresyl sulfate) [19], microbiome-derived metabolites [20] and dysregulated hormones involved in CKD-mineral bone disorder (CKD-MBD) [21]. Consequently, it is possible that phosphate became an independent factor only in some of the models we analyzed, since other uremic toxins might be responsible for the rest. Moreover, we did not discover significant differences in the nutritional status, either in serum albumin or MNA scores, between those with and without VC (Table 1). Judging from our findings, the gustatory function modulates the probability of VC among CKD/ESRD patients likely through altering the levels of multiple procalcific uremic toxins instead of altering the nutritional status at its entirety. However, we did not measure the serum calcification propensity, which may be a more comprehensive indicator of VC status [22], in our participants. On the other hand, a better gustatory function may serve as a surrogate for other factors that influences the probability of having VC, such as taste receptor activities in other organs [23]. Nonetheless, more evidence is needed to uncover the intricate connection between gustatory function and uremic VC.

DM has been shown to impair gustatory function in the existing literature [24]. However, we believe that DM might not be responsible for the relationship between gustatory dysfunction and uremic VC observed in this study. First, though there is a trend of differences in the prevalence of DM between those with and without VC (Table 1), we already adjusted for DM in the logistic regression analyses (Table 3). Second, we further examined the degree of glycemic control between groups with and without VC. There was no difference between diabetic patients with and without VC regarding their glycated hemoglobin levels (with vs. without, 7.0 ± 1.0 vs. 6.7 ± 0.9%, *p* = 0.36). Based on these results, we think that the association between the gustatory function and the risk of VC was not modified or mediated by DM.

Among the four taste categories examined, we found that only bitter taste exhibited a close relationship with the probability of VC (Table 3). Apart from the influence introduced by the overall gustatory function, bitter taste may have its unique property that distinguishes itself from other taste categories. Prior studies indicated that bitter taste was preferentially compromised during chemotherapy-induced taste alterations relative to salt and sweet tastes [25]. The alterations in bitter tastes may influence the preferred choices of food, such as proteinaceous ones, among affected individuals [26]. In addition, the relationship between altered bitter taste and VC may be related to the activities of bitter taste receptors and inflammatory cytokines [27,28]. Nonetheless, there are still much uncertainty with regards to the explanation for the relationship we observed. More mechanistic work is needed.

The clinical implications of our findings can be broad. For clinicians caring for patients with CKD/ESRD, the preserved gustatory function empowers them to have better nutrition and improve their quality of life; however, there can be downsides, according to the results we presented. Special attention needs to be devoted to their dietary choices, since their blood vessels can be adversely influenced by uremic toxins generated from what they ingest. A follow-up of the calcification severity involving major vessels may be an underrecognized component of care for CKD/ESRD patients with a preserved gustatory function.

Our study has its strengths and limitations. The strengths include the novelty of our findings, which have not been reported before and potentially shed light on the need for VC monitoring among a subgroup of patients with renal function impairment. The extensive variables we collected and analyzed also decreased the possibility of residual confounders for our results. Nonetheless, several limitations should be noted before extrapolating these findings. First, our cohort size was modest, limiting the statistical efficiency of the analyses. Second, we did not measure other types of uremic toxins, such as protein-bound ones, so we cannot affirm whether these unmeasured uremic toxins modify the risk of VC in these patients. In addition, we did not have these patients record their dietary composition to calculate the phosphate content. Finally, we only checked AAC in this study without checking other vascular segments, including abdominal aorta or lower extremity arteries; however, according to our prior study, the consistency of VC severity between different anatomical areas was good among our cohort of CKD/ESRD patients [29].

## 4. Conclusions

In conclusion, based on a prospectively assembled cohort of CKD patients, we showed that higher aggregate taste strip scores, or a better gustatory function, was associated with an increased probability of having uremic VC. Furthermore, serum phosphate partially accounted for the observed relationship, signifying the importance of uremic toxins in the pathogenesis of VC. Larger studies with longitudinal follow-ups of great vessels among patients with CKD/ESRD may be required for reaching a definite conclusion.

## 5. Materials and Methods

### 5.1. Cohort Establishment

We prospectively enrolled adult patients (age ≥ 20 years) with an estimated glomerular filtration rate (eGFR) <60 mL/min/1.73 m^2^ stably for at least three months from the nephrology clinics in National Taiwan University Hospital Beihu Branch between 2017 and 2018. Exclusion criteria consisted of those who refused to provide written informed consent, those without posteroanterior chest roentgenographic films on enrollment and those with diseases that obviously impaired gustatory function (oral or upper respiratory infections and inflammatory bowel disease). For participants with a malignancy or prior cerebrovascular disease, we excluded those who received prior irradiation or had any sequel of cranial nerve palsy. After enrollment, we collected their demographic data and comorbidity profile through interview and performed physical examinations to obtained anthropometric parameters, including BP, body mass index and waist circumference. Following physical examination, participants received blood tests for hemogram and serum biochemistry, including nutrition, electrolyte panels, renal function and lipid profiles. Spot urine was also obtained and analyzed for estimating daily proteinuric amounts.

Since gustatory function is closely associated with appetite and food section [30], we also assessed participants’ appetite statuses and nutritional competency using the CNAQ and MNA toolkit, respectively. CNAQ, a simple 8-item questionnaire created decades ago, has a score range between 8 (worst) and 40 (best) for assessing appetite. Those with a score <28 were deemed to have prominent appetite loss [31]. Results based on CNAQ have been shown to predict the risk of weight loss and malnutrition in adults and institutionalized patients, and its results correlate closely with findings using other instruments [31]. MNA was also introduced more than 2 decades ago as a validated systematic nutritional screening instrument for many different populations [32]; we used the screening part of the MNA, which consisted of 6 items with a score range between 0 (severely malnourished) and 14 (normal), for analysis in this study. Those with a score <12 were recognized as at risk of or having frank malnutrition [33].

### 5.2. Exposure Characterization

Gustatory function status is the main exposure in this study. We evaluated participants’ gustatory functions using the following two approaches: the objective one based on a well-established and validated taste strip kit (Burghart Medizintechnik, Wedel, Germany) and the subjective one based on self-reported data (taste intactness and taste importance) rated on a visual analog scale with scores between 0 and 100. For the objective approach, we tested 4 taste categories: sweet, sour, salty and bitter, each of which was assessed by corresponding solute-impregnated paper strips of 4 graded concentrations (scores 1 to 4 for each taste). Participants were blinded regarding taste strip sequences and had mouth washes between each test. For each taste category, a category-specific taste score and the proportion of participants with a defect involving such taste (score < 2) were also obtained. After adding up scores of the 4 taste categories, an aggregate taste strip score (range 4 to 16) and an aggregate taste category count (range 0 to 4) were obtained for each participant.

### 5.3. Outcome Measurement

VC was the main outcome of this study. We measured VC based on a semi-quantitative AAC scoring system using the posteroanterior chest films, according to prior studies and reviews [34,35]. This scoring system used to be tested and applied to our patients with CKD as well [29,36]. Participants were divided into those with and without AAC in the subsequent analyses.

### 5.4. Statistical Analysis

Continuous variables were shown in means with standard deviations, while categorical variables were described as numbers with percentages. Variables first underwent tests for whether they complied with a normal distribution using the Kolmogorov-Smirnov test. Normally distributed continuous variables were compared by the Student’s *t*-test, and non-normally distributed ones were compared by the Mann-Whitney U test. Categorical variables were analyzed using the chi-square test.

We first compared differences with regards to clinical features and laboratory data between those with and without AAC. Gustatory function parameters, including the aggregate taste strip scores, taste category counts and those for each taste category; the proportions of participants with defects in each taste category; the subjective evaluation of taste intactness and the perceived importance of taste integrity, were compared between groups with and without AAC using appropriate tests. This was followed by multiple regression analyses with taste strip scores, taste category counts and individual taste categories that differed according to the AAC status as the dependent variable, incorporating variables exhibiting a *p*-value < 0.1 between groups in the univariate analysis. The basic model considered demographics, comorbidities, anthropometric parameters and laboratory data selected from the univariate analysis only, while the other model additionally included the serum phosphate level. VIF results were used to examine the issue of collinearity. All statistical analyses were done using SPSS 19th version. A *p*-value < 0.05 was deemed statistically significant.

### 5.5. Ethical Approval

Our study protocol adhered to the Declaration of Helsinki and was approved by the institutional review board of National Taiwan University Hospital (No. 201710041RIND; Date: 6 December 2012). We obtained written inform consent from all participants.

## Figures and Tables

**Table 1:** Clinical features of chronic kidney disease patients with and without AAC.

	Total (*n* = 58)	With AAC (*n* = 35)	Without AAC (*n* = 23)	*p*-Value
Demographic profile				
Age (years)	72.4 ± 10.9	75 ± 7.5	68.6 ± 14.1	0.030
Sex (male %)	38 (65.5)	22 (62.9)	16 (69.6)	0.607
Comorbidities				
Diabetic mellitus (%)	31 (53.5)	22 (62.9)	9 (39.1)	0.079
Hypertension (%)	47 (81.0)	31 (88.6)	16 (69.6)	0.073
Cirrhosis (%)	1 (1.7)	1 (2.9)	0 (0)	0.422
Coronary artery disease (%)	2 (3.5)	2 (5.7)	0 (0)	0.251
Prior myocardial infarction (%)	2 (3.5)	2 (5.7)	0 (0)	0.251
Heart failure (%)	1 (1.7)	0 (0)	1 (4.4)	0.220
Atrial fibrillation (%)	3 (5.2)	2 (5.7)	1 (4.4)	0.822
Chronic obstructive pulmonary disease (%)	1 (1.7)	1 (2.9)	0 (0)	0.422
Rheumatologic illnesses (%)	3 (5.2)	1 (2.9)	2 (8.7)	0.335
Malignancy (%)	7 (12.1)	4 (11.4)	3 (13.0)	0.857
Peptic ulcer disease (%)	7 (12.1)	6 (17.1)	1 (4.4)	0.857
Prior cerebrovascular disease (%)	7 (12.1)	6 (17.1)	1 (4.4)	0.148
Anthropometric parameters				
Systolic blood pressure (mmHg)	129.6 ± 15.6	131.3 ± 16.2	126.8 ± 14.3	0.283
Heart rate (/min)	73.8 ± 10	74.5 ± 9.7	72.9 ± 10.6	0.559
Body height (cm)	162.2 ± 8.4	161.3 ± 8.4	163.5 ± 8.4	0.348
Body weight (kg)	65.8 ± 11.6	64.6 ± 10.8	67.6 ± 12.7	0.346
Body mass index (kg/m^2^)	24.9 ± 3.5	24.8 ± 3.6	25.1 ± 3.5	0.718
Waste circumference (cm)	90.0 ± 10.6	90.7 ± 9.0	88.9 ± 12.7	0.523
Laboratory data				
Hemoglobin (mmol/L)	7.3 ± 1.2	7.1 ± 1.1	7.6 ± 1.2	0.191
Platelet (K/μL)	212.7 ± 71.6	208.6 ± 71.7	218.8 ± 72.6	0.602
Leukocyte (K/μL)	6.4 ± 1.9	6.1 ± 1.9	6.9 ± 2.0	0.119
Albumin (g/L)	41.0 ± 3.1	41.0 ± 2.8	41.0 ± 3.6	0.939
Urea nitrogen (mmol/L)	14.4 ± 6.5	15.1 ± 5.2	13.4 ± 8.1	0.353
Creatinine (μmol/L)	288.4 ± 357.2	316.5 ± 402.6	245.6 ± 277.4	0.465
eGFR (mL/min/1.73 m^2^)	32.0 ± 15.7	29.8 ± 15.8	35.3 ± 15.2	0.197
Sodium (meq/L)	137.8 ± 2.7	137.5 ± 2.8	138.3 ± 2.5	0.246
Potassium (meq/L)	4.5 ± 0.5	4.6 ± 0.5	4.4 ± 0.4	0.175
Calcium (mmol/L)	2.3 ± 0.1	2.3 ± 0.1	2.3 ± 0.2	0.448
Phosphate (mg/dL)	3.8 ± 0.7	3.9 ± 0.7	3.6 ± 0.7	0.082
Uric acid (μmol/L)	362.4 ± 110.7	360.6 ± 120.0	365.3 ± 97.5	0.875
Total cholesterol (mmol/L)	4.3 ± 0.9	4.5 ± 0.9	4.1 ± 0.9	0.147
Triglyceride (mmol/L)	1.6 ± 0.9	1.7 ± 0.9	1.4 ± 0.8	0.191
Spot urine protein-creatinine ratio	0.75 ± 0.97	0.86 ± 1.04	0.59 ± 0.85	0.300
Appetite and nutritional status				
CNAQ scores	27.9 ± 3.8	28.3 ± 4.0	27.3 ± 3.5	0.334
Anorexia (based on CNAQ)	23 (39.7)	13 (37.1)	10 (43.5)	0.637
MNA-SF scores	12.9 ± 1.6	12.7 ± 1.8	13.1 ± 1.3	0.334
Malnutrition (based on MNA-SF)	11 (19.0)	7 (20.0)	4 (17.4)	0.808

AAC, aortic arch calcification; CNAQ, Council on Nutrition Appetite Questionnaire; eGFR, estimated glomerular filtration rate based on the Modification of Diet in Renal Disease (MDRD) formula and MNA-SF, Mini Nutritional Assessment—screening form.

**Table 2:** Gustatory function results of chronic kidney disease patients with and without AAC.

	Total (*n* = 58)	With AAC (*n* = 35)	Without AAC (*n* = 23)	*p*-Value
Taste strip aggregate score				0.039
0 ~ 3	24 (41.4)	11 (31.4)	13 (56.5)	
4 ~ 6	19 (32.8)	11 (31.4)	8 (34.8)	
7 ~ 13	15 (25.9)	13 (37.1)	2 (8.7)	
Taste strip intact category counts				0.046
0	25 (43.1)	11 (31.4)	14 (60.9)	
1 ~ 2	20 (34.5)	13 (37.1)	7 (30.4)	
3 ~ 4	13 (22.4)	11 (31.4)	2 (8.7)	
Defective sweet taste (%)	37 (63.8)	20 (57.1)	17 (73.9)	0.2
Sweet taste scores				0.523
0 ~ 2	51 (87.9)	30 (85.7)	21 (91.3)	
3 ~ 4	7 (12.1)	5 (14.3)	2 (8.7)	
Defective sour taste (%)	35 (60.3)	18 (51.4)	17 (73.9)	0.09
Sour taste scores				0.361
0 ~ 2	50 (86.2)	29 (82.9)	21 (91.3)	
3 ~ 4	8 (13.8)	6 (17.1)	2 (8.7)	
Defective salty taste (%)	40 (69.0)	21 (60.0)	19 (82.6)	0.071
Salty taste scores				0.069
0 ~ 1	40 (69.0)	21 (60.0)	19 (82.6)	
2 ~ 3	18 (31.0)	14 (40.0)	4 (17.4)	
Defective bitter taste (%)	42 (72.4)	22 (62.9)	20 (87.0)	0.045
Bitter taste scores				0.045
0 ~ 1	42 (72.4)	22 (62.9)	20 (87.0)	
2 ~ 4	16 (27.6)	13 (37.1)	3 (13.0)	
Subjective taste intactness (0–100)	81.5 ± 14.4	79.9 ± 17.0	83.9 ± 8.9	0.298
Subjective taste importance (0–100)	84.1 ± 16.1	85.0 ± 14.2	82.8 ± 18.9	0.619

AAC, aortic arch calcification.

**Table 3:** Multiple logistic regression analyses with the presence of vascular calcification as the dependent variable.

	Odds Ratio	95% CI	*p*-Value
Model 1: Basic model *, including aggregate taste strip scores			
Age (per year)	1.096	1.026–1.171	0.006
Hypertension	6.564	1.295–33.277	0.023
Taste strip scores (per score)	1.288	1.022–1.623	0.032
Model 2: Basic model *+P, including aggregate taste strip scores			
Age (per year)	1.104	1.028–1.184	0.006
Hypertension	5.300	1.070–26.259	0.041
Taste strip scores (per score)	1.284	1.017–1.622	0.036
Model 3: Basic model *, including taste intactness category count			
Age (per year)	1.089	1.022–1.161	0.009
Hypertension	6.529	1.348–31.609	0.020
Taste strip intact category (per category)	1.923	1.152–3.211	0.012
Model 4: Basic model *+P, including taste intactness category count			
Age (per year)	1.089	1.022–1.161	0.009
Hypertension	6.529	1.348–31.609	0.020
Phosphate (mg/dL)	1.923	1.152–3.211	0.012
Model 5: Basic model *, including bitter taste scores			
Age (per year)	1.104	1.031–1.183	0.005
Hypertension	8.435	1.510–47.101	0.015
Bitter taste score (per score)	2.558	1.167–5.606	0.019
Model 6: Basic model *+P, including bitter taste scores			
Age (per year)	1.115	1.034–1.203	0.005
Hypertension	7.333	1.279–42.056	0.025
Bitter taste score (per score)	2.656	1.215–5.806	0.014
Phosphate (mg/dL)	3.205	1.079–9.522	0.036
Model 7: Basic model *, including proportions with defective bitter taste			
Age (per year)	1.082	1.018–1.149	0.011
Hypertension	5.997	1.248–28.827	0.025
Defective bitter taste	0.175	0.036–0.847	0.030
Model 8: Basic model *+P, including proportions with defective bitter taste			
Age (per year)	1.100	1.028–1.177	0.006
Hypertension	5.167	1.029–25.936	0.046
Defective bitter taste	0.168	0.032–0.879	0.035

CI, confidence interval and P, serum phosphate. * Including age, gender, hypertension and diabetes mellitus.

## Abbreviations

AAC	aortic arch calcification
CI	confidence interval
CKD	chronic kidney disease
CKD-MBD	chronic kidney disease-mineral bone disorder
CNAQ	Council on Nutrition Appetite Questionnaire
DM	diabetes mellitus
eGFR	estimated glomerular filtration rate
ESRD	end-stage renal disease
MNA	Mini-Nutritional Assessment
OR	odds ratio
VC	vascular calcification
VSMC	vascular smooth muscle cell
